# Type I interferon response gene expression in established rheumatoid arthritis is not associated with clinical parameters

**DOI:** 10.1186/s13075-016-1191-y

**Published:** 2016-12-12

**Authors:** Tamarah D. de Jong, Marjolein Blits, Sander de Ridder, Saskia Vosslamber, Gertjan Wolbink, Mike T. Nurmohamed, Cornelis L. Verweij

**Affiliations:** 1 Amsterdam Rheumatology and Immunology Center, VU University Medical Center, CCA 2.21, P.O. Box 7075, 1007MB Amsterdam, The Netherlands; 2 Department of Pathology, VU University Medical Center, Amsterdam, The Netherlands; 3 Amsterdam Rheumatology and Immunology Center, Reade, Amsterdam, The Netherlands

**Keywords:** Rheumatoid arthritis, Type I interferon, Gene expression

## Abstract

**Background:**

A peripheral blood interferon (IFN) signature (i.e., elevated type I interferon response gene [IRG] expression) has been described in a subset of patients with rheumatoid arthritis (RA). In the present study, we systematically assessed the association between this IRG expression and clinical parameters.

**Methods:**

Expression of 19 IRGs was determined in peripheral blood from 182 consecutive patients with RA and averaged into an IFN score per individual. Correlation and unpaired analyses were performed on the complete patient group. The analyses were internally validated by using an algorithm to randomize the patient group 1000 times into two equally sized sets, and then analyses were performed on both sets.

**Results:**

Associations were assessed between IFN score and disease duration, 28-joint Disease Activity Score and its components, the occurrence of erosions and nodules, autoantibody positivity, and immunosuppressive treatment. This analysis revealed lower IFN scores in patients using hydroxychloroquine, prednisone, and/or sulfasalazine, but it did not show significant associations between the other parameters and the IFN score. Selecting patients who were not treated with hydroxychloroquine, prednisone, and/or sulfasalazine (*n* = 95) did not reveal any significant associations either.

**Conclusions:**

IRG expression in RA is affected by immunosuppressive treatment with prednisone, hydroxychloroquine, and/or sulfasalazine, but it is not evidently associated with other clinical parameters. Hence, the IFN signature appears to describe a subgroup of patients with RA but does not seem to reflect disease activity.

**Electronic supplementary material:**

The online version of this article (doi:10.1186/s13075-016-1191-y) contains supplementary material, which is available to authorized users.

## Background

Rheumatoid arthritis (RA) is a systemic autoimmune disease characterized by chronic joint inflammation. It manifests as a heterogeneous disease that is partly reflected at the level of gene expression. Genome-wide gene expression analysis revealed evidence for molecular differences between patients with RA, in particular in the type I interferon (IFN) system. Approximately 50% of patients with RA display a peripheral blood IFN signature (i.e., relatively high expression of interferon response genes [IRGs]) [1].

Type I IFNs were initially known for their antiviral effects, but increasing insight into their activities revealed their role as pleiotropic cytokines with a critical role in modulating immune responses, such as cellular activation, major histocompatibility complex upregulation, induction of apoptosis, and inhibition of angiogenesis [2]. It is thought that type I IFNs contribute to autoimmunity by initiating a break of tolerance (e.g., by the induction of dendritic cell maturation and inhibition of regulatory T cells) [3]. The exact role of the IFN signature in RA is yet unknown, although it was shown to have potential clinical relevance. That is, (1) the presence of the IFN signature was shown to be a risk factor for arthritis development in preclinical disease [4], and (2) the presence of the IFN signature in established RA was found to be associated with the clinical response to treatment with rituximab [5] and tocilizumab [6].

Earlier studies have addressed whether the IFN signature in RA could be associated with clinical parameters, which inconclusively revealed a potential relationship of the IFN signature with anticitrullinated protein antibody (ACPA) titers [7, 8]. However, these study cohorts were rather small (35 subjects or less) and therefore highly subject to a lack of power. Hence, the relationships between the peripheral blood IFN signature and disease- and inflammation-related clinical parameters have never been thoroughly assessed. In the present study, we used a larger cohort of patients with established RA (*n* = 182) in combination with a random sampling algorithm to systematically investigate whether the peripheral blood IFN signature in RA could be associated with parameters such as disease activity, laboratory parameters, and the use of immunosuppressive treatment.

## Methods

### Patient recruitment and blood collection

Patients with RA (*n* = 182) were consecutively recruited from the Jan van Breemen Research Institute, Reade center, Amsterdam, The Netherlands. All patients were Caucasian and were diagnosed with RA according to the American College of Rheumatology 1987 criteria [9]. Ninety-five percent of the patients displayed a 28-joint Disease Activity Score (DAS28) of ≥3.2 despite earlier treatment with at least two disease-modifying antirheumatic drugs (DMARDs). All patients provided written informed consent, and this study was approved by the medical ethics committee of Reade. The patients’ characteristics are shown in Table 1. From all patients, 2.5 ml of blood was drawn into PAXgene tubes (PreAnalytiX GmbH, Hombrechtikon, Switzerland) and stored at −20 °C until further processing.

**Table 1:** Patient characteristics

	All patients (*n* = 182)
Demographic parameters	
Age, years, mean (SD)	54.2 (11.8)
Female sex, *n* (%)	135 (75)
Disease parameters	
Disease duration in years, mean (SD)	9.7 (10.3)
DAS28, mean (SD)	5.1 (1.2)
Erosive disease, *n* (%)	131 (72)
Nodules, *n* (%)^a^	43 (24)
Laboratory parameters	
ESR, mm/h, mean (SD)	24.5 (18.0)
CRP, mg/L, mean (SD)	17.8 (22.1)
IgM-RF titer, IU/ml, mean (SD)^b^	124.7 (279)
IgM-RF-positive, *n* (%)^b^	95 (59)
ACPA titer, AU/ml, mean (SD)^c^	1563 (2680)
ACPA-positive, *n* (%)^c^	131 (75)
IFN score, mean (SD)	0.26 (1.01)
Medication parameters	
MTX use, *n* (%)	152 (84)
MTX dosage in mg/week, mean (SD)	21.0 (6.3)
Prednisone use, *n* (%)	52 (29)
Prednisone dosage in mg/day, mean (SD)	7.2 (3.5)
HCQ use, *n* (%)	35 (19)
SSZ use, *n* (%)	27 (15)

*Abbreviations: ACPA* Anticitrullinated protein antibodies, *CRP* C-reactive protein, *DAS28* 28-joint Disease Activity Score, *ESR* Erythrocyte sedimentation rate, *HCQ* Hydroxychloroquine, *IFN* Interferon *IgM-RF* Immunoglobulin M rheumatoid factor, *MTX* Methotrexate, *SSZ* Sulfasalazine

^a^Not available for 6 patients

^b^Not available for 21 patients

^c^Not available for 7 patients

### RNA isolation, complementary DNA synthesis, and real-time polymerase chain reaction

Total RNA was isolated from the PAXgene tubes according to the manufacturer’s instructions. The quantity and purity of the RNA samples were checked using a NanoDrop spectrophotometer (Thermo Scientific, Wilmington, DE, USA). We reverse-transcribed 0.25 μg of RNA into complementary DNA (cDNA) using a RevertAid H Minus cDNA Synthesis Kit (Thermo Fisher, Waltham, MA, USA). A single aliquot of each cDNA sample was first subjected to 14 cycles of specific target amplification using a 0.2× mixture of all TaqMan gene expression assays in combination with the TaqMan PreAmp Master Mix (Applied Biosystems, Foster City, CA, USA). Following preamplification, the samples were diluted 1:5 (vol/vol) in Tris-ethylenediaminetetraacetic acid buffer, pH 8.0. Multiplex real-time quantitative polymerase chain reaction was performed using the 96.96 Biomark Dynamic Array systems (Fluidigm Corporation, South San Francisco, CA, USA) at ServiceXS (Leiden, The Netherlands) according to the manufacturer’s instructions. Quantities were calculated relative to GAPDH using the standard curve method. Expression levels were log_2_-transformed.

### Calculation of the IFN score and statistical analyses

Nineteen IRGs described to be components of the IFN signature in RA [1] were measured (see Additional file 1: Table S1). Log_2_-transformed expression levels of the IRGs were highly correlated (*r* ≥ 0.7 for 90% of the combinations, *p* ≤ 0.002); therefore, an IFN score was calculated by averaging these values of all genes for each sample.

Data were analyzed using IBM SPSS Statistics version 22 (IBM, Armonk, NY, USA), R version 3.1.3 [10], and GraphPad Prism version 5.01 (GraphPad Software, La Jolla, CA, USA) software. For internal cross-validation, a 1000-times random sampling method was used to randomize the group of 182 patients into 2 equally sized sets and to execute Spearman correlation for continuous variables and Mann-Whitney *U* analysis for dichotomous variables on each set [11]. *p* Values <0.05 were considered to be significant. Correction for multiple testing was performed using the method of Benjamini and Hochberg.

## Results

We studied the association between the peripheral blood IFN score and the following parameters: disease duration, DAS28 and its individual components, the occurrence of erosions and nodules, autoantibody positivity, and immunosuppressive treatment. As demonstrated in Table 2, prednisone use and dose, hydroxychloroquine (HCQ) use, and sulfasalazine (SSZ) use were the only variables that showed a significant result, of which only HCQ use remained significant after correction for multiple testing. Similar results were obtained in the cross-validation using the random sampling algorithm, which revealed significance for HCQ use only: a *p* value <0.05 was detected in both sets in 521 of the 1000 iterations, in 1 of the 2 sets for 479 of 1000 iterations, and never in none of the sets (median *p* value 0.015). A slight trend was also observed for prednisone use and dose and SSZ use (median *p* values 0.090–0.14, median coefficient for prednisone dose −0.18), although significance was never found in both sets for these variables. Each IRG was also analyzed individually, which revealed similar results (data not shown).

**Table 2:** Analysis of associations between interferon score and clinical parameters after 1000-times random sampling

	Complete group (*n* = 182)		Cross-validation				
	*p* Value	BH-corrected *p* value	Significant results (*p* < 0.05)			Median *p* values	
			Both sets	One set	Neither set	Set 1	Set 2
Disease parameters							
Disease duration	0.061	0.25	0	371	629	0.18	0.20
DAS28	0.18	0.41	0	199	801	0.38	0.32
TJC28	0.10	0.36	0	264	736	0.25	0.25
SJC28	0.61	0.76	1	39	960	0.53	0.59
VAS	0.21	0.44	0	122	878	0.39	0.35
Erosions	0.41	0.60	0	61	939	0.50	0.51
Nodules	0.24	0.46	1	143	856	0.39	0.41
Laboratory parameters							
ESR	0.86	0.98	6	18	976	0.60	0.58
ESR dichotomous (>20)	0.71	0.85	0	19	981	0.60	0.58
CRP	0.14	0.39	0	233	767	0.30	0.29
CRP dichotomous (≥10)	0.15	0.38	0	190	810	0.30	0.33
RF titer	0.36	0.60	1	66	933	0.46	0.50
RF positivity	0.96	1.0	3	3	994	0.62	0.61
ACPA titer	0.38	0.59	0	64	936	0.47	0.51
ACPA positivity	0.86	0.98	1	9	990	0.64	0.64
ACPA high positivity (≥3× cutoff)	0.29	0.52	0	79	921	0.44	0.44
RF- and ACPA-positive vs. rest	0.57	0.79	0	34	966	0.59	0.58
RF- and ACPA-negative vs. rest	0.13	0.41	0	219	781	0.27	0.28
Medication parameters							
MTX use			2	3	995	0.66	0.65
MTX dosage	0.57	0.79	1	29	970	0.58	0.58
Prednisone use	0.037	0.19	0	526	474	0.14	0.14
Prednisone dosage	0.017	0.14	0	718	282	0.092	0.090
HCQ use	0.001	0.013	521	479	0	0.015	0.015
SSZ use	0.023	0.14	0	584	416	0.11	0.11
PREDN and/or HCQ and/or SSZ use	0.00032	0.0080	612	388	0	0.012	0.010

*Abbreviations: BH* Benjamini-Hochberg, *HCQ* Hydroxychloroquine, *SSZ* Sulfasalazine, *PREDN* Prednisone, *ACPA* Anti-citrullinated protein antibodies, *CRP* C-reactive protein, *DAS28* 28-joint Disease Activity Score, *DMARD* Disease-modifying antirheumatic drug, *ESR* Erythrocyte sedimentation rate, *RF* Rheumatoid factor, *MTX* Methotrexate, *SJC28* Swollen joint count in 28 joints, *TJC28* Tender joint count in 28 joints, *VAS* Visual analogue scale

**Fig. 1:**
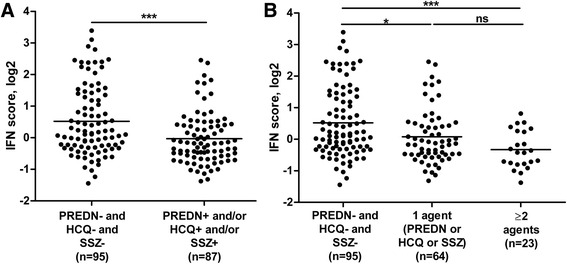
Comparison of interferon (IFN) scores between patients with different disease-modifying antirheumatic drugs and prednisone (PREDN) treatments. IFN scores were compared between patients who were not treated with PREDN, hydroxychloroquine (HCQ), and sulfasalazine (SSZ) and patients treated with one or more of those agents. Data from the complete cohort (*n* = 182) are displayed. **a** Patients divided into treated or not with one or more of the three agents. **b** Patients subdivided into treated with none of the three agents, one of the three agents, or two or more of the three agents. * *p* ≤ 0.05, *** *p* ≤ 0.001. *ns* Not significant

For prednisone, HCQ, and SSZ treatment, the IFN score was lower in the treated group than in the untreated group. Combining HCQ use, prednisone use, and SSZ use also revealed a significantly lower IFN score in patients using HCQ and/or prednisone and/or SSZ than in patients not treated with any of these agents, both in the analysis of the complete cohort and in the cross-validation (*p* = 0.0080 with Benjamini-Hochberg correction, median cross-validation *p* ≤ 0.012) (see Table 2 and Fig. 1a). Moreover, the suppressive effect appeared larger for patients treated with two or more of those agents than for patients treated with one agent (Fig. 1b). No association was found between IFN score and methotrexate (MTX) treatment or dose. The relation between IFN score and treatment did not appear to be confounded by DAS28 or disease duration.

Because the suppression of IFN score in HCQ-, prednisone-, and/or SSZ-treated patients could have a masking effect on other associations between IFN signature and clinical parameters, we also performed the analyses for the selection of patients who were not treated with prednisone, HCQ, and/or SSZ (*n* = 95) at the moment of blood collection. This did not result in any significant associations between the IFN score and other variables (uncorrected *p* value ≥0.099, median *p* value cross-validation ≥0.23) (see Additional file 1: Table S2).

## Discussion

The present study is the first use of a systematic approach in a relatively large cohort to study the relationship between the IFN signature in established RA and clinical parameters. We demonstrated that the IFN signature was suppressed in patients treated with HCQ, prednisone, and/or SSZ, but not with MTX. Furthermore, we did not observe any associations between the IFN signature and the other clinical parameters.

Van der Pouw Kraan et al. showed that a subgroup of patients with RA displays a common pathogen-response program, which was characterized by a higher incidence of the IFN signature as well as higher ACPA titers, suggesting that these parameters might be associated with one another [7]. However, a causal relationship was not established, and our data indicate that this is not the case. The IFN signature was not significantly different between ACPA-negative and ACPA-positive patients, nor did it significantly correlate with ACPA titers. Possibly, the IFN signature and ACPA positivity are independently associated with activation of the common pathogen response program, because they are both implied to be induced via certain pathogens [12, 13].

Our cohort consisted mainly of established patients with RA with a DAS28 ≥ 3.2 despite treatment with at least two DMARDs. Although our data do not suggest any association between IFN score and DAS28 (*p* > 0.1 in all analyses), we cannot fully exclude the possibility that significant associations could have been found if the cohort had included more patients with low disease activity. Inclusion of patients with inactive and/or early disease could provide a more complete view of the IFN response in different states of the disease.

Remarkably, the IFN scores were decreased in HCQ-, prednisone-, and/or SSZ-treated patients, even though the beneficial effects of these treatments were supposedly diminished. Moreover, cotreatment with these agents appeared to have an additive suppressive effect. Interference of both prednisone and HCQ with type I IFN signaling has been described before [14, 15], but the influence of SSZ remains to be elucidated. It has been shown that SSZ reduces the levels of RA-related cytokines, such as interleukin-1β and tumor necrosis factor-α [16], suggesting that SSZ might function through overall suppression of inflammatory cytokines, including type I IFNs. Furthermore, it was demonstrated that SSZ is able to accelerate apoptosis of neutrophils [17], which we have recently shown to be major inducers of the type I IFN response in RA [18]. Consequently, suppression of the type I IFN response by SSZ might be mediated via an increase in neutrophil apoptosis.

As previously described, suppression of the IFN score by certain treatment could affect the applicability of the IFN signature as a biomarker for therapy response, particularly to rituximab [5, 19]. That is, the treatment-related suppression of IFN score might impair the discriminative capacity of the biomarker, which would consequently lead to more false predictions. Because multiple studies have demonstrated that the extent of the IFN signature is highly variable between patients [1, 20, 21], we considered it important to assess the IFN score as a continuous variable rather than as a dichotomous variable. However, inclusion of a healthy control population would allow us to determine whether patients with prednisone, SSZ, and/or HCQ treatment would still display an IFN response above the levels of healthy control subjects. This could also give more insight into the applicability of the IFN response as a biomarker in these patients. Future studies should be done to elucidate the effect of each individual treatment, as well as combinatory therapy, on the IFN signature and the corresponding response prediction. Alternatively, presence of the IFN signature in individuals with arthralgia was shown to be associated with a higher risk for developing arthritis [4]. It would be interesting to study whether early treatment with one of the implied suppressors of the IFN response could delay or even prevent disease onset.

## Conclusions

Our data indicate that there are no evident associations between the peripheral blood IFN signature in established RA and clinical parameters. This suggests that the IFN signature is not an indication of disease activity per se, but its presence could indicate a potential difference in pathology or immune pathway activation compared with patients without this signature. Consequently, this could influence the response to therapy, particularly to biologics because these are specific modulators of these immune pathways.

## Additional file

Additional file 1: Table S1. List of the 19 IFN response genes measured. **Table S2.** Results of analysis for associations between IFN scores and clinical parameters after 1000 times of random sampling using only patients who were not treated with prednisone, HCQ, or SSZ. (PDF 82 kb)

## Abbreviations

ACPA: Anticitrullinated protein antibodies
BH: Benjamini-Hochberg
cDNA: Complementary DNA
CRP: C-reactive protein
DAS28: 28-joint Disease Activity Score
DMARD: Disease-modifying antirheumatic drug
ESR: Erythrocyte sedimentation rate
HCQ: Hydroxychloroquine
IFN: Interferon
IgM-RF: Immunoglobulin M rheumatoid factor
IRG: Interferon response gene
MTX: Methotrexate
PREDN: Prednisone
RA: Rheumatoid arthritis
SJC28: Swollen joint count in 28 joints
SSZ: Sulfasalazine
TJC28: Tender joint count in 28 joints
VAS: Visual analogue scale
