# Supplementary material for: Type I interferon response gene expression in established rheumatoid arthritis is not associated with clinical parameters
Source: Arthritis Res Ther. 2016 Dec 12;18:290. doi: 10.1186/s13075-016-1191-y (PMC5154062; doi:10.1186/s13075-016-1191-y)
Supplement: Additional file 1: Table S1. — List of the 19 IFN response genes measured. Table S2. Results of analysis for associations between IFN scores and clinical parameters after 1000 times of random sampling using only patients who were not treated with prednisone, HCQ, or SSZ. (PDF 82 kb) [file 13075_2016_1191_MOESM1_ESM.pdf]

SUPPLEMENTARY TABLES

**Table S1** List of the 19 IFN response genes measured	
**Gene Symbol**	**Gene Name**
EPSTI1	epithelial stromal interaction 1
HERC5	HECT and RLD domain containing E3 ubiquitin protein ligase 5
IFI35	interferon-induced protein 35
IFI44	interferon-induced protein 44
IFI44L	interferon-induced protein 44-like
IFI6	interferon, alpha-inducible protein 6
IFIT1	interferon-induced protein with tetratricopeptide repeats 1
IFITM1	interferon induced transmembrane protein 1
IL1RN	interleukin 1 receptor antagonist
ISG15	ISG15 ubiquitin-like modifier
LGALS3BP	lectin, galactoside-binding, soluble, 3 binding protein
LY6E	lymphocyte antigen 6 complex, locus E
MX1	MX dynamin-like GTPase 1
MX2	MX dynamin-like GTPase 2
OAS1	2'-5'-oligoadenylate synthetase 1, 40/46kDa
OAS2	2'-5'-oligoadenylate synthetase 2, 69/71kDa
RSAD2	radical S-adenosyl methionine domain containing 2
SAMD9L	sterile alpha motif domain containing 9-like
SERPING1	serpin peptidase inhibitor, clade G (C1 inhibitor), member 1

Table S2 Analysis results of associations between IFN score and clinical parameters after 1000 times of random sampling using only patients that were not treated with prednisone, HCQ or SSZ.

	Complete group (n=95)	Cross-validation				
		Significant results (p<0.05)			Median p values	
	P value	Both sets	One set	Neither set	Set 1	Set 2
**Disease parameters**						
Disease duration	0.63	2	13	985	0.61	0.59
DAS28	0.30	0	122	878	0.42	0.46
TJC28	0.099	0	256	744	0.23	0.27
SJC28	0.98	12	15	973	0.62	0.61
VAS	0.61	4	27	969	0.57	0.55
Erosions	0.26	0	107	893	0.43	0.40
Nodules	0.23	1	129	870	0.40	0.39
**Laboratory parameters**						
ESR	0.79	4	19	977	0.59	0.58
ESR dichotomous (>20)	0.69	2	14	984	0.60	0.59
CRP	0.59	0	41	959	0.56	0.55
CRP dichotomous (>10)	0.77	2	8	990	0.60	0.59
RF titer	0.56	2	49	949	0.54	0.55
RF positivity	0.65	1	30	969	0.59	0.59
ACPA titer	0.63	2	27	971	0.59	0.59
ACPA positivity	0.74	0	12	988	0.62	0.62
ACPA high positivity (≥3x cutoff)	0.19	0	126	874	0.33	0.39
RF and ACPA positive vs. rest	0.86	3	18	979	0.63	0.64
RF and ACPA negative vs. rest	0.33	0	53	947	0.47	0.48
**Medication parameters**						
MTX use	0.94	1	4	995	0.66	0.65
MTX dosage	0.63	1	20	979	0.60	0.60
